# A bias of Asparagine to Lysine mutations in SARS-CoV-2 outside the receptor binding domain affects protein flexibility

**DOI:** 10.3389/fimmu.2022.954435

**Published:** 2022-12-09

**Authors:** Jennifer C. Boer, Qisheng Pan, Jessica K. Holien, Thanh-Binh Nguyen, David B. Ascher, Magdalena Plebanski

**Affiliations:** ^1^ School of Health and Biomedical Science, Royal Melbourne Institute of Technology, Melbourne, VIC, Australia; ^2^ School of Chemistry and Molecular Biosciences, University of Queensland, Brisbane, QLD, Australia; ^3^ Computational Biology and Clinical Informatics, Baker Heart and Diabetes Institute, Melbourne, VIC, Australia; ^4^ School of Science, Royal Melbourne Institute of Technology (RMIT) University, Melbourne, VIC, Australia

**Keywords:** molecular modelling, Omicron, infectiousness, mutation, SARS-CoV-2

## Abstract

**Introduction:**

COVID-19 pandemic has been threatening public health and economic development worldwide for over two years. Compared with the original SARS-CoV-2 strain reported in 2019, the Omicron variant (B.1.1.529.1) is more transmissible. This variant has 34 mutations in its Spike protein, 15 of which are present in the Receptor Binding Domain (RBD), facilitating viral internalization via binding to the angiotensin-converting enzyme 2 (ACE2) receptor on endothelial cells as well as promoting increased immune evasion capacity.

**Methods:**

Herein we compared SARS-CoV-2 proteins (including ORF3a, ORF7, ORF8, Nucleoprotein (N), membrane protein (M) and Spike (S) proteins) from multiple ancestral strains. We included the currently designated original Variant of Concern (VOC) Omicron, its subsequent emerged variants BA.1, BA2, BA3, BA.4, BA.5, the two currently emerging variants BQ.1 and BBX.1, and compared these with the previously circulating VOCs Alpha, Beta, Gamma, and Delta, to better understand the nature and potential impact of Omicron specific mutations.

**Results:**

Only in Omicron and its subvariants, a bias toward an Asparagine to Lysine (N to K) mutation was evident within the Spike protein, including regions outside the RBD domain, while none of the regions outside the Spike protein domain were characterized by this mutational bias. Computational structural analysis revealed that three of these specific mutations located in the central core region, contribute to a preference for the alteration of conformations of the Spike protein. Several mutations in the RBD which have circulated across most Omicron subvariants were also analysed, and these showed more potential for immune escape.

**Conclusion:**

This study emphasizes the importance of understanding how specific N to K mutations outside of the RBD region affect SARS-CoV-2 conformational changes and the need for neutralizing antibodies for Omicron to target a subset of conformationally dependent B cell epitopes.

## Introduction

SARS-CoV-2 is part of the *Betacoronavirus* genus, a highly diverse group of viruses characterized by positive-sense, single-strand RNA (1), which can infect many mammalian and avian species. SARS-CoV-2 infection occurs mainly *via* the Spike protein (2–4), which is structurally characterized by an S1 subunit and an S2 subunit. The S1 contains the receptor binding domain (RBD), while the S2 region drives the membrane fusion (5).

Binding of the human ACE2 receptor to RBD is a critical step for initiation of target cell entry and can occur with high affinity even at very low molar ranges of viral proteins (6). The RBD undergoes conformational changes that fluctuate between configurations identified as “up” (or open) and “down” (or closed) states. The “up” configuration allows accessibility to the ACE2 receptor binding site, while in the “down” state it remains hidden (4, 7). In addition, the binding of RBD to ACE2 receptor exposes the viral S2 domain allowing it to insert the fusion peptide into the target cell membrane (8, 9). Studies like these are a strong indication that residues outside of the Spike protein RBD area can also play a critical role in viral pathogenesis and underpin the abilty of the virus to dock onto host cells.

As the rapid evolution of SARS-CoV-2 continues, new variants like Omicron (B.1.1.529.1 or BA.1) and its subvariants (BA.2, BA.3, BA.4, BA.5, BQ.1 and BBX.1) have emerged, which contain an alarming number of Spike protein mutations. A total of 34 mutations have been identified in the Spike protein of Omicron, when compared to the original Wuhan strain (10). Mutations are mostly found in the RBD and N-terminal domain, which at the same time, are also both major targets for neutralizing antibodies. The high number of mutations present in Omicron is also a great cause of concern for the efficacy of existing vaccines as well as immune-therapeutics and has led Pfizer to formulate a new vaccine targeted against Omicron, which passed Phase III trials (11). Many attempts have been made to better understand the SARS-CoV-2 neutralizing antibody binding properties and how these have evolved to compromise protection provided by vaccination or prior infection. For instance, through the use of an artificially constructed neutralization resistant virus expressing the Omicron Spike protein variant, authors showed that Omicron and the neutralization resistant Spike construct, were both 30-180 fold more resistant to neuralization by convalescent plasma, compared to the original Wuhan sequence (12).

Meanwhile, a molecule-based data-driven type of analysis compared the binding free energy (BFE) of Omicron against Wuhan RBD complexes, to 132 known antibody specificities (13). The results showed that the mutations present in Omicron had a considerable impact on antibody binding to the virus and suggested an ongoing natural evolutionary pressure of the SARS-CoV-2 virus to direct its antigenic drift towards evading human immune response. Most importantly, the authors concluded that the emerging Omicron mutations, enable the virus to escape antibody immunity induced by current vaccines (13).

The repercussions of an evolutionary pressure that directs specific point mutations of SARS-CoV-2 toward antibody evasion is of considerable importance and requires robust investigation. Understanding the mutations that could affect conformational stability, antibody docking and recognition of these types of B cell epitopes, is imperative for successful vaccine design, and will help foster strategies able to promote effective production of neutralizing antibodies in response to vaccination, correlated with long-term protective immunity against viral infection.

In this paper, we investigated mutations specifically occurring in Omicron and it’s subvariants, across several proteins when compared to all the other prominent ancestral variants. Of these mutations, a vast majority appeared to be an N to K mutation occurring specifically in the Spike protein region. Although the N to K mutations occur mainly outside of the RBD region, they are potential key contributors to the change of the RBD conformations of the Spike protein. Further to that, several mutations in the RBD of Omicron’s subvariants were also analysed, which showed a stronger potential of immune escape, compared to the prototype. These results emphasize the understanding of how mutations outside of the RBD area can affect structural organization of the virus and can help further our knowledge of B cell epitope recognition, which is crucial for the advancement of peptide-based future vaccine design strategies.

## Materials and methods

In this work, we first performed the alignments on the full-spectrum of SARS-CoV-2 proteins of all the current and past VOCs, followed by computational structural analysis to investigate the effects of residue mutation specifically on Omicron and its subvariants. Additional *in-silico* experiments were performed on the mutations in RBD to explore the infectiousness and immune escape properties of Omicron Spike variants directly.

### SARS-CoV-2 variant alignment

For SARS-CoV-2 variants protein alignment, we used the original Omicron, 5 different subvarariants, two emerging variants and the original Wuhan sequence (**Table 1**). The FASTA sequences were retrieved from the GISAID database (https://www.gisaid.org/) (14–16). In the EpiCov search section of GISAID there is an available tab that allows for the selection of the major circulating variants. Of these, we selected the current VOC Omicron (including its subvariants BA.1, BA.2, BA.3, BA.4, BA.5 plus XBB.1 and BQ.1, the two variants predicted to emerge as dominant variants) and previously circulating VOCs (Alpha, Beta, Gamma, and Delta). The virus names listed below are GISAID nomenclature and the specific viruses were selected based on various conditions: for all variants we selected the conditions in which the sequence was complete and excluding sequences with low coverage. Variants were chosen based on their historical appearance. The specific amino acid sequences of the various genomic regions were obtained from the selected variants with FASTA sequence on GISAID and searched using BlastN (17, 18). Within the cross-platform sequence alignment editor Jalview (19), we performed Multiple Sequence Alignment using Fast Fourier (MAFFS) (20) which is a high-speed multiple sequence alignment algorithm utilizing the Fast Fourier Transform to optimize protein alignments based on the amino acidic physical properties (19). We further aligned the amino acid sequences for the surface glycoprotein, membrane protein and nucleoprotein, ORF3a, ORF7 and ORF8 areas of Alpha, Beta, Gamma, Delta and Omicron variants plus the original Wuhan sequence, and set the latter as a reference genome.

**Table 1:** SARS-CoV-2 prototype and Variants of Concern.

Nr	Virus name GISAID nomenclature	Equivalent NCBI accession nr	Clade	Lineage	% sequence Identity GISAID vs NCBI	Variant
1	hCoV-19/Wuhan/WIV04/2019	MN996528.1	Original	Original	100%	Wuhan
2	hCoV-19/England/205041766/2020	MZ005945.1	FRY	B1.1.7	99%	Alpha
3	hCoV-19/Japan/IC-0564/2021	MW988204.1	GR	P.1	100%	Gamma
4	hCoV-19/SouthAfrica/KRISP-EC-K004574/2020	MW981442.1	GH	B.1.351	99%	Beta
5	hCoV-19/India/MH-NCCS-BJ1/2021	MZ023220.1	G	B1.1617	99%	Delta
6	hCoV-19/USA/NM-CDC-QDX32337620/2021	OM202878.1	GRA	B.1.1.529.1	99%	Omicron
7	hCoV-19/Botswana/R165B92_BHP_AAC32282/2021	ON375778.1	GRA	BA.1	99%	Omicron
8	hCoV-19/USA/CA-ASC-210844543/2022	ON080219.1	GRA	BA.2	99%	Omicron
9	hCoV-19/Denmark/DCGC-392185/2022	OP170269.1	GRA	BA.3	99%	Omicron
10	hCoV-19/USA/NY-Wadsworth-22042624-01/2022	OP147180.1	GRA	BA.4	99%	Omicron
11	hCoV-19/Denmark/DCGC-588045/2022	OX278505.1	GRA	BA.5	100%	Omicron
12	hCoV-19/Malaysia/IMR_OS6350/2022	ON674677.1	GRA	BA.2.12.1	99%	Omicron
13	hCoV-19/Australia/NSW-ICPMR-35588/2022	OP661948.1	GRA	BA.2.75	100%	Omicron
14	hCoV-19/USA/AZ-ASU92993/2022	OP607549.1	GRA	BQ.1	99%	Omicron
15	hCoV-19/India/TN-CDFD-O-162/2022	OP659449.1	GRA	BBX.1	99%	Omicron

### Spike protein structure curation *via* homology modelling

To build the complete structure for the following *in-silico* structural analysis, homology modelling was performed to build the missing regions in the experimental structures using MODELLER version 10.2 (21). We built the Spike protein trimer to obtain a comprehensive 3D insight, rather than only model the crucial region, such as the RBD (22–24). The different templates used to model were listed in **Table 2**.

**Table 2:** The templates in homology modelling.

Variant	Form	Binding partner	Template (PDB code)	RMSD to template (Å)
Prototype	down (closed)	apo	7DWY^*^ (27)	0.34
Prototype	up (open)	apo	7CZZ^*^ (28), 7KRR (29)	0.69
Prototype	up (open)	ACE2	7KJ4^*^ (30), 7KRR (29)	1.92
Prototype	up (open)	P5A-2F11 (antibody)	7CZZ^*^ (28,27), 7KRR	1.27
Omicron	down (closed)	apo	7DWY^*^ (27)	0.50
Omicron	up (open)	apo	7CZZ^*^ (28), 7KRR (29)	0.72
Omicron	up (open)	ACE2	7KJ4^*^ (30), 7KRR (29)	1.29
Omicron	up (open)	P5A-2F11 (antibody)	7CZZ^*^ (28), 7KRR (29)	1.18

^*^All atom RMSD was calculated based on this template.

Two apo trimeric Spike protein systems, all RBD-up and all RBD-down, were prepared for this work. Alignment for modelling Omicron Spike protein was based on protein sequence change in Omicron to reduce human artefacts (25). For the RBD-down system, we employed a cryo-EM-determined structure with three RBD being down state (PDB ID: 7DWY) (26) to model the Spike folding. For the RBD-up system, we used the Spike-Ab complex having all monomers in up position with missing region from residues 827 to 854 (PDB ID: 7CZZ) (27) and Spike protein (PDB ID: 7KRR) (28) having only 1 monomer in up position (chain A) without missing region from residues 827 to 854 as templates. Similar to the modelling of the apo Spike RBD-up system, the Spike-ACE2 and Spike-Ab models were built using the template complex Spike-ACE2 (PDB ID: 7KJ4 (29) and Spike-Ab (PDB ID: 7CZZ), respectively. The missing region from both templates were built using up conformation of Spike monomer (PDB ID: 7KRR).

The model with lowest Discrete Optimized Protein Energy (DOPE) score (30) was chosen. Structural difference was measured using all-atom Root mean square deviation (RMSD) calculated by superimposing the homology models with the main templates (PDB ID: 7DWY, 7CZZ, and 7KJ4) for different systems (**Table 2**). Our models showed low structural deviation with the experimental structures (**Table 2**) and the AlphaFold2 models (RMSD: 0.55 Å) (22). Final models for Spike protein with both open and closed conformation were available in the **Supplementary Materials** for detailed comparison. The PyMol Molecular Graphics System, Version 2.0 (31), was used to visualise the protein structures and generate the figures.

### Mutation analysis

Mutational tolerance was explored using the COVID-3D resource (32).

The effects of the mutations on protein thermodynamic stability and dynamics were calculated using SDM (33), mCSM-Stability (34), DUET (35), ENCoM (36), mCSM-membrance (37), DynaMut (38), and DynaMut2 (39). The effects of the mutations on the protomers interaction as well as the Spike-ACE2 interaction were evaluated using mCSM-PPI (34), mCSM-PPI2 (40), and mmCSM-PPI (40); which have been previously shown to correlate strongly with experimental data on this complex (41). The effects of the mutations on antibody binding were analysed using the protein models described above of the S protein bound to the monoclonal antibody from COVID-19 convalescent patients P5A-2F11 (PDB:7CZZ). These calculations were conducted using mCSM-AB (42), mCSM-AB2 (43), and mmCSM-AB (44). Each method outputs the change in Gibb’s free energy (ΔΔG) of thermodynamics or affinity (in Kcal/mol). The inputs of these structure-based predictors were the homology models described previously. Since these homology models are more structurally similar to the actual experimental structures, these we utilized instead of adopting the snapshots derived by our MD. This provides more accurate and informative results in a single, easy to compare value.

All atomic MD simulations of the prototype and Omicron variants in the open and closed forms were performed using GROMACS (version 2020) for 20 ns in triplicate. Amber ff99SB-ILDN (45) force field and TIP3P water model (46) were applied to the systems. Detailed information on MD simulations can be found here (47). In short, the system was neutralized and solvated in a periodic cubic box with its wall being 1nm away from the complex atoms. The system was first minimized for 50,000 steps using the steepest descent algorithm, followed by the equilibration over 100 ps each at the constant volume and the constant pressure of 1 atm. Weak harmonic positional restraints on the complex atoms with a force constant were imposed during the minimization and these initial equilibration steps. The system was then carried out for 20 ns at 300 K in the constant pressure (NPT) ensemble in triplicate without the constraints of all the complex atoms. Hydrogen bond (Hbond) interaction was analysed using a cutoff distance of 3.5 Å. C-alpha RMSD for Spike-ACE2 and Spike-Ab of prototype and Omicron Spike proteins were calcualted to monitor the simulation (**Figure S6**).

## Results

### Multiple sequence alignment between VOCs in SARS-CoV-2

**Figure 1:**
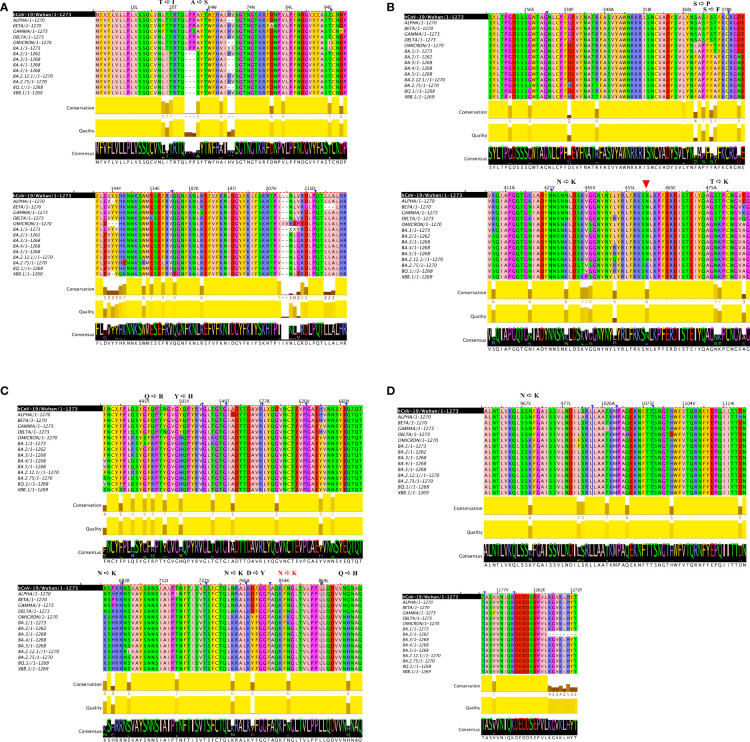
Multiple alignments in 6 VOC strains. Multiple sequence alignment of full-length Spike proteins of Alpha, Beta, Gamma, Delta and Omicron SARS-CoV-2 VOCs including it’s subvariants (BA.1, BA.2, BA.3, BA.4, BA.5) as well as currently emerging variants (BQ.1 and BBX.1) show a distinct preference for N to K transition in the Spike region. **(A)** is first portion of the Spike protein **(B)** second portion of the Spike protein, red arrow indicates another NtoK transition acquired **(C)** third portion of the Spike protein, NtoK in red indicates a lost mutation **(D)** final portion of the Spike protein. In order to facilitate localization of where mutations have occurred, in **(A-C)** a truncated Spike protein is depicted, blue arrows and lines indicate the sites where a portion of the amino acid sequence is not visible. Full length Omicron protein with annotated mutations can be viewed in **Figure S1A–C**.

**Figure 2:**
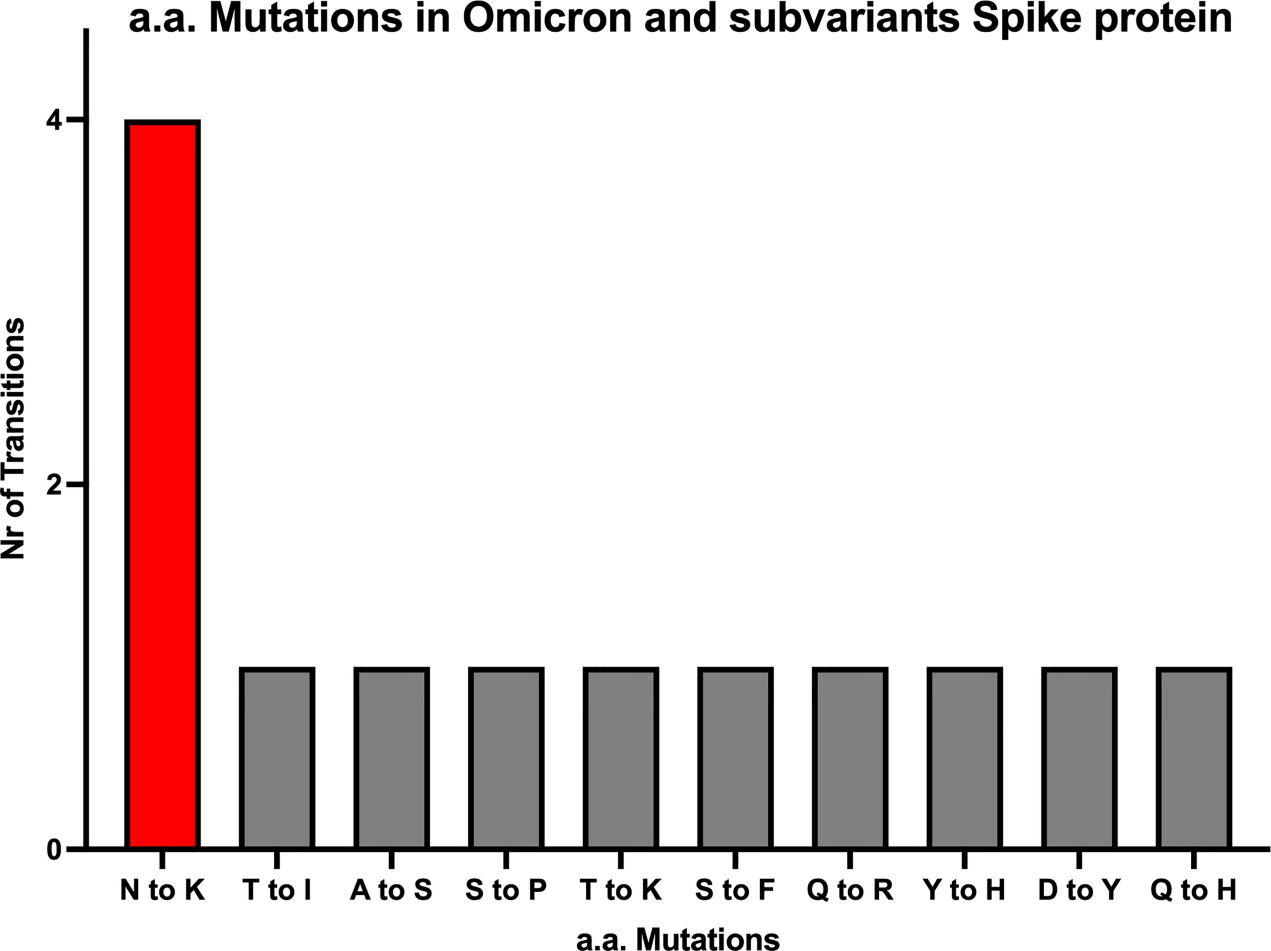
All amino acid mutations in Omicron Spike protein. The numbers of N to K mutations are about 4-fold higher compared to 9 other mutations occurring in the SARS-CoV-2 Spike region of Omicron and it’s subvariants.

Despite the high numbers of mutations present in Omicron, evidence from phylogenetic trees, has so far shown no intermediate branches of evolution when comparing Omicron to previous VOCs (48, 49). When investigating the alignments we specifically examined the ones that showed amino acid conservation across all other variants and exhibited change only in Omicron. A total of 34 mutations are present in Omicron. Of these, there are 24 mutations consisting in amino acid mutations that have occurred only in Omicron (**Figure 1** and **Figures S1A–C**) whilst remaining consistent throughout the other variants. Of the 24 Omicron specific mutations, 14 are specific to Omicron as well as all its subvariants. Of these 14, four are N to K mutations (30%), while the remaining mutations only occurred in one (7,6%) instances (**Figure 2**). Interestingly when looking at other SARS-CoV-2 regions of interest like N, ORF3a, ORF7 and ORF8 (**Figure S2–5**), none of these regions showed a specific bias towards N to K mutations, indicating that although it is occurring outside of the RBD region, this genetic variation is still highly specific to the Spike protein sequence.

Furthermore, missense tolerance ratio (MTR) of all 30 missense mutations reported in Omicron Spike protein were obtained using COVID-3D tools (**Table S1**). Only three mutations (A67V, G446S, and L981F) were located at intolerant positions, indicating that they were not under purifying selection.

### Mutation analysis on three N to K mutations on the thermodynamics stability and protomer interaction of Spike protein apo structure

Given the highly skewed N to K mutations taking place in the Spike protein area, we next investigated these specific mutations. Both N764K and N969K mutations were highly conserved in all the Omicron subvariants (BA.1 - BA.5) and emerging subvariants (BQ.1, BBX.1), while N856K was only reported in the main variant (BA.1) (**Figures 1A–D**). Both N440K and N679K have previously been described, with N440K located in RBD reported to affect the interaction of Spike protein with antibody (50), and N679K likely to increase the virus infection by enhancing the cleavage of S1 and S2 subunits (51). Thus, we focussed on the remaining three N to K mutations (N764K, N856K and N969K), which are all located in the central core region of the S2 subunit of Spike protein, to explore the potential molecular consequences on the change of RBD conformation.

These three mutations, N764K, N856K, and N969K, were predicted to mildly destabilise the structure by mCSM-Stability, SDM, DUET, ENCoM, DynaMut1, mCSM-Membrance (single-site mutation predictions, **Table S2**), and DynaMut2 (multiple-site mutation predictions, **Tables 3**, **S2**), consistent with the prediction of I-Mutant 3.0 (52). Previous work (53) has shown that using homology models as inputs for these predictors was reliable with DynaMut2 showing the best consistency. Furthermore, only DynaMut2 accepts multiple-site mutations. Thus, only the results from this predictor were presented in the main text. Only N856K in open conformation has a mild positive prediction on the Spike protein. The degree of deleterious effects on Spike thermodynamics stability was slightly stronger on open conformation of Spike (down:up = -1.87 Kcal/mol:-2.00 Kcal/mol, average on all protomers, **Table 3**), indicating that the weaker destabilisation of the closed conformations would potentially account for the preference of RBD conformation by these three N to K mutations.

**Table 3:** The effects of Omicron multiple-site mutations on protein thermostability and stability of the trimeric Spike protein.

Mutations	Protomer ID	DynaMut2 (Kcal/mol) - closed conformation	DynaMut2 (Kcal/mol) - open conformation	mmCSM-PPI (Kcal/mol) - closed conformation	mmCSM-PPI (Kcal/mol) - open conformation
N764K	A/B/C	-1.62	-1.89	-0.60	-0.72
N856K	A/B/C	-1.75	0.85	-1.57	-2.28
N969K	A/B/C	-1.9	-1.86	-0.60	-0.48
N764K/N856K/N969K	A	-1.86	-2.05	-0.70	-0.79
N764K/N856K/N969K	B	-1.83	-1.93	-0.84	-1.12
N764K/N856K/N969K	C	-1.93	-2.02	-1.05	-0.73

The effects of these three mutations on the affinity of the trimeric Spike was also evaluated by measuring how the global stability on the Spike complex contributes to the interaction between each protomer. All three mutations showed a comparable destabilising effect on the Spike trimer when either the open or closed conformation was analysed (down:up = -0.86 Kcal/mol:-0.88 Kcal/mol, average on all protomers, **Table 3**). Single-site mutation effect predictions are displayed in the (**Table S3**). Of all N to K mutants, N856K mutation of both the open and closed conformations showed the strongest destabilising effect.

**Figure 3:**
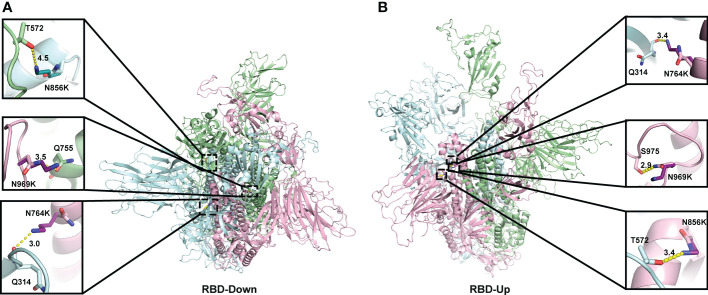
Location of three N to K mutations in the central core region of the homology model of Spike protein. The monomers of the Spike proteins in open **(A)** and closed **(B)** were shown in ribbons with different colours (pink, green, cyan). Side chain atoms of both prototype and Omicron residues were shown in sticks with the Carbon atoms in light and dark colours, respectively. The residues making Hydrogen bond interactions with prototype and Omicron residues are shown in sticks. Hydrogen bond interaction was shown in yellow dash line. The zoom-in versions of the interactions were shown next to the Spike protein.

When analysing the structural changes, for the N764K mutation, the side chain of K764 in the model of both the open and closed conformations, forms an additional hydrogen bond with Q314 (**Figures 3A–B**). For N856K, the side chain of K856 in Omicron model in open conformation forms an additional hydrogen bond with T572 (**Figure 3B**). Both of these are in agreement with previous studies (10, 54, 55). The similar polar contacts between protomers may account for similar mutation effects on two conformations.

### Mutation effects on the Spike-ACE2 complex

Four mutations (S477N, Q493R, Q498R, and N501Y) located on the binding surface of RBD in the Spike protein, which were nominated based on previous studies (55) and were consistently observed in different Omicron subvariants, were identified by mCSM-PPI, mCSM-PPI2, and mmCSM-PPI to have mild effects on the affinity of the interaction between Spike protein and human ACE2 receptor protein (**Table 4**). While Q493R and Q498R were predicted to decrease binding affinity, consistent with the introduction of a larger charged residue, S477N and N501Y were predicted to stabilise the interaction. The calculation of the change of binding affinity caused by N501Y is also consistent with a previous MD study (56).

**Table 4:** Mutation effects on the Spike-ACE2 complex.

Mutations	Distance to surface (Å)^*^	mCSM-PPI (Kcal/mol)	mCSM-PPI2 (Kcal/mol)	mmCSM-PPI (Kcal/mol)	Outcome
S477N	5.63	0.42	0.12	0.12	Increased affinity
Q493R	2.85	-1.86	-0.71	-0.71	Decreased affinity
Q498R	3.18	-2.86	-1.11	-1.11	Decreased affinity
N501Y	3.43	-1.79	0.52	0.52	Increased affinty
S477N/Q493R/Q498R/N501Y	/	/	/	-0.92	Descreased affinity

^*^Distance to surface is a measurement from the wild-type residue to the binding interface between Spike and ACE2 in the homology model, to indicate the biochemical property of the mutation, which is not used to measure of the binding.

**Figure 4:**
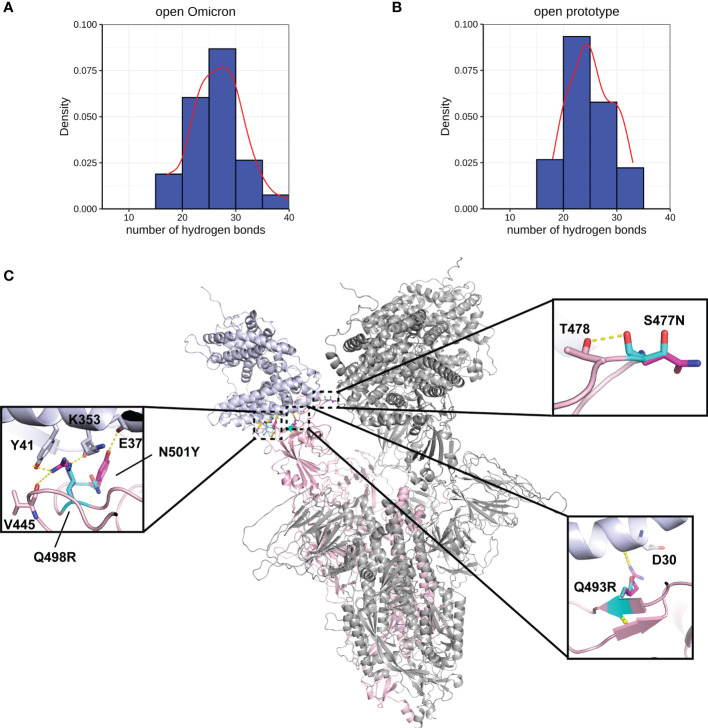
Interactions between Spike and ACE2. Hydrogen bond interaction distribution between residues in Spike (residues 332-527) and ACE2 proteins during 20 ns of triplicate MD simulations in **(A)** open Omicron and **(B)** open prototype variants are shown in blue histogram with its smoothed density line shown in red. These both represent RBD-up conformation of Omicron and Prototype Spike proteins. Density on y axis refers to the Kernel density, the image depicts the probability density function of the variable. The Spike protein was presented in ribbon with three RBD all binding to ACE2 **(C)**. Four mutations in the receptor binding interface were zoom-in. Both prototype (cyan) and Omicron residues (magenta) were shown in sticks with the Carbon atoms on Spike-ACE2 complex (pink-blue). Hydrogen bond was shown in yellow dash line.

A more comprehensive study on multiple-site mutation effects was examined using mmCSM-PPI. This also supported the mild change of Spike-ACE2 binding. The new polar contacts formed by the Omicron mutant residues, including R493 (Spike) with D30 (ACE2), R498 (Spike) with Y41 and K353 (ACE2), and Y501 (Spike) with E37 (ACE2), can provide potential explanations for this (**Figure 4C**). To further explore this, we ran a short MD simulation on both the prototype and Omicron Spike and analysed the effect of each variant on Spike-ACE2 complexes. The distribution of the number of Hbond interactions between Spike (residues 332-527) and ACE2 proteins (**Figures 4A, B**) was measured and shown that the open form of Omicron has majority of the number of Hbond interactions 25 to 30, while the prototype has majority of the number of Hbond interactions between 20 and 25. It showed that Omicron has a stronger interaction in majority of the cases comparing to the prototype. This observation is consistent with our structural predictions (**Table 4**) where few of the mutations increase the binding affinity between Omicron and ACE2.

### Mutation effects on the Spike-antibody complex

Another four mutations (S477N, T478K, E484A, and Q493R), located on the RBD-antibody interface, were nominated based on previous works (57, 58) and were repetitively observed in different Omicron subvariants. P5A-2F11 is one of the neutralizing monoclonal antibodies derived from the COVID-19 convalescent patients (27), which presents strong compatibility with ACE2. The effects of the mutations on the recognition by the P5A-2F11 antibody were analysed as a representative to better understand the immune evasion of Omicron, especially for repetitive positive cases reported in the post-COVID age.

**Figure 5:**
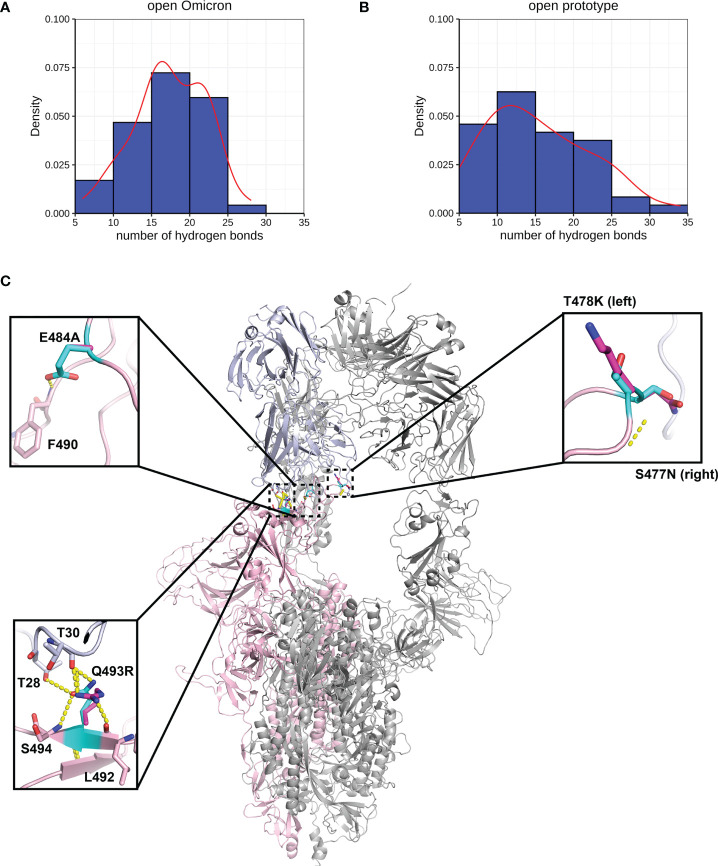
Interactions between Spike and antibody (P5A-2F11). Hydrogen bond interaction distribution between residues in Spike (residues 332-527) and antibody proteins during 20 ns of triplicate MD simulations in **(A)** Omicron and **(B)** prototype variants are shown in blue histogram with its smoothed density line shown in red. Both represent RBD-up conformation of Omicron and Prototype Spike proteins. Density on y axis refers to the Kernel density, the image depicts the probability density function of the variable The Spike protein was presented in ribbon with three RBD all binding to P5A-2F11 **(C)**. Both prototype (cyan) and Omicron residues (magenta) were shown in sticks with the Carbon atoms on Spike-P5A-2F11 complex (pink-blue). Hydrogen bond was shown in yellow dash line.

We performed MD of these systems and obtained snapshots of the Spike proteins. The distribution of the number of Hbond interactions between Spike (residues 332-527) and antibody proteins (**Figures 5A, B**) were measured and showed that the prototype has more portion of Hbond interaction from 25 to 35 than the Omicron. Although the difference is small because of the short MD simulations, it showed that Omicron has a tendency to have weaker interaction to AB than the prototype. All of these four mutations, located at the Spike-P5A-2F11 interface, were individually predicted by mCSM-AB, mCSM-AB2, and mmCSM-AB to decrease the binding affinity of the complex. These four individual mutations were predicted to mildly reduce the binding between Spike and P5A-2F11, (**Table 5**) consistent with previous *in-silico* work on epitopes (59), while the combination of these mutations was predicted to have a much larger reduction in recognition by P5A-2F11, consistent with earlier work on other neutralizing antibodies (10). The deteoriation of Spike-P5A-2F11 binding is likely due to the change of polar contacts of these mutants (**Figure 5C**). Our results indicated the mutations reported in the Omicron subvariants could potentially decrease the neutralizing effect of antibodies, even on patients recovering from the infection, which could underpin the observed connection between the high infectiousness and immune escape properties of Omicron.

**Table 5:** Mutation effects on the Spike-Ab complex.

Mutations	Distance to surface (Å)^*^	mCSM-AB (Kcal/mol)	mCSM-AB2 (Kcal/mol)	mmCSM-AB (Kcal/mol)	Outcome
S477N	2.91	0.80	-0.01	-0.14	Decreased affinity
T478K	4.88	-0.11	-0.32	-0.15	Decreased affinity
E484A	4.01	-0.59	-0.42	-0.02	Decreased affinity
Q493R	2.7	0.76	-0.39	-0.49	Decreased affinity
S477N/T478K/E484A/Q493R	/	/	/	-0.80	Decreased affinity

^*^Distance to surface is a measurement from the wild-type residue to the binding interface between Spike and Ab in the homology model, to indicate the biochemical property of the mutation, which is not used to measure of the binding.

## Discussion

Understanding the evasion of humoral responses by viruses and the critical consequences for antibody immunotherapies as well as vaccine design, are extremely important for the identification of novel treatments. As the SARS-CoV-2 virus continues its ever-changing journey, it becomes increasingly important to unravel the complex molecular aspects of increased transmissibility as well as the viral modality of genetic drift.

Much focus has been dedicated to investigating key mutations, including the mutations of K417 and E484 in RBD (60), and dominant mutations like the N501Y and D614G (61), which present themself across a majority of variants. Others have even looked at antibody evasion properties of Omicron subvariants (62) as well as the use of deep mutational scanning to identify mutationally constrained areas in the RBD regions, which represent ideal targets for antibodies (63, 64). Further to that, it has been established that the RBD constitutes a key functional component of the S1 subunit, responsible for SARS-CoV-2 binding to lung cells through ACE2 (6, 55–58). Although a highly pursued avenue has been studying the combination of various mutations (65), to date there haven’t been studies in which the authors have investigated a skew towards one type of specific mutation, like the N to K mutations, and specifically investigated the viral properties solely in this context.

Using a range of bioinformatic tools, here we showed for the first time a skew towards N to K mutation both inside and outside the RBD region, but only present in the Spike protein of SARS-CoV-2. Out of the four N to K mutations, three (N764K, N856K, and N969K) are located in the trimerization region and exhibit mild negative contributions to the protein folding and the interactions of Spike protomers. Interestingly, we also noticed that these three N to K mutations on the three RBD-down conformation are more energetically stable, suggesting the trimeric Omicron Spike protein with all RBD-down state may benefit from these residue substitutions. This may subsequently promote the escape of recognition from antibodies. Furthermore, the stronger mild destabilising effects caused by mutations for RBD-up conformation may potentially provide the flexibility for the Spike structure. The negative effects on Spike thermodynamics stability caused by these three N to K mutations were consistent with the predictions from I-Mutant 3.0 (52). The missing loop from residue 824 to 854 in our homology models was structurally similar to the cryo-EM determined structure (66), but some local residue environment might still differ from ones determined from experimental structures (10, 55), presenting a larger molecular distance between K856 and T572 in the closed conformation (**Figure 3A**).

The RBD undergoes changes in the conformation that can either expose the binding site or not. These “up” and “down” conformational alterations pose an interesting problem for SARS-CoV viruses as immune recognition is less efficient when RBD is hidden in a down conformation compared to when it is exposed. Conversely hidden RBD may lead to inefficient host cell interaction and host cell entry. Previous studies using cryo-EM structures and constant-pH Monte Carlo simulations showed that enhanced virulence could also be a consequence of an improved viral stability of the trimeric Spike in the open state with the better RBD availability to ACE2, rather than only through the alteration in the RBD-ACE2 interaction itself (10, 67, 68). Meanwhile, more research revealed that the Spike protein of Omicron is more likely to have one RBD-up conformation, not only maintaining the interaction with ACE2 but also restricting the recognition of antibody (69, 70). In our *in-silico* work, we identified that three N to K mutations reported in Omicron and its subvariants may contribute to a mild preference of all the RBD-down conformation, compared to the prototype Spike protein. Although our results were not directly comparable with the previous studies, both showed that the ever-changing COVID Spike protein may adopt a strategy to restrict less RBD-up conformation to facilitate immune evasion (71). We showed a new insight of the Omicron mutations outside RBD which could contribute to this conformational alteration. Two of these three N to K mutations (N764K and N969K) have been circulating in Omicron BA.1 to the latest dominant BA.5, and we, hence, expected this N to K bias may be kept in the new dominant variant, which attracts attention on the dynamics of the Spike protein.

The preference of the alteration of RBD conformation is crucial for the availability of the interaction with ACE2 and antibody. The change of the binding of Spike-ACE2 and Spike-Ab upon Omicron mutations in RBD, however, play a vital role on the infection of Omicron. Four different mutations on the binding interface of Spike-ACE2 and Spike-Ab were investigated according to the distance of the wild-type residue to the interface and previous studies. In addition, from our alignments, we observed that the selected mutations remain consistent in all the subsequently emerged variants to date. Previous studies informed a stronger binding between Omicron Spike protein and ACE2 (23, 56, 72) and a reduction of binding between Omicron Spike protein and Abs (52, 58, 70). The predictive increased binding affinity caused by N501Y was consistent with these findings, while the other calculations may slightly differ. However, we based our results on homology models, which may vary from the actual experimental structures. The prototype Spike-ACE2 model has the largest structural difference (**Table 2**) with the experimental one. Since the prediction tools used in our study are sensitive to the input structure (53), these factors may affect the measurement of the change of binding. Overall there have been substantial studies on antibody neutralization effect of Omicron subvariants (73–75). We further selected one Spike-Ab, derived from convalescent patient as a proof of principle. Previous studies have extensively identified individual mutation patterns without focusing on specific amino acid mutations (10, 52, 55, 60–62, 64, 65). So far, the N to K mutation N440K is the only one located within the RBD area of the Omicron variant. For instance, SARS-CoV-2 Omicron G339D and N440K mutations are located in a neighbouring site called antigenic site IV, which in turn is a known recognition site by the S309 mAb (76). Interestingly, previous studies have shown that the Lysine side chain introduced by the N440K substitution does not affect binding of S309 (77), while others have demonstrated that K417N mutation both alone or in combination with other mutations, produces a greater ACE2 affinity than a K417T mutation either alone or in combination with other mutations (60).

Another important aspect to consider is whether this genetic drift could potentially be the result of gene editing inherent to the virus rather than an evolutionary pressure driven by circulating vaccines. Interestingly, the predilection of N to K mutations over other mutations cannot be explained by viral RNA editing enzymes like Adenosine Deaminases that Act on RNA (ADARs). ADARs are RNA editing enzymes that play an important role in regulating transcriptome and proteome diversity. This type of editing can have important roles that function in favour or against viral survival and can even change over the course of an infection (78). However, ADARs are known to exhibit a preference for adenosine to inosine (A to I) transition, where the inosine modification will subsequently be read as guanosine (G). Therefore, when looking at the Asparagine (AAT and AAC) and Lysine (AAG and AAA) codons, as both last codons of Asparagine are not an A, the Asparagine to Lysine (N to K) variation is unlikely due to ADAR preference in mutation. It is, therefore, more likely that these genetic variations are vaccine-driven rather than a mutational preference in the viral replication machinery.

In our studies, this emphasized the importance of using molecular dynamics (MD) simulations and computational mutation analysis methods to understand SARS-CoV-2 evolution, since antigenic drifting could have large implications to the preference of RBD conformation, which is associated with studies on B cell epitopes and vaccine design. A particular point of concern for Omicron is its phylogenetically different lineage which is highly distinct from all the previously dominant SARS-CoV-2 variants. Although the mutations on the RBD may not significantly improve the binding of ACE2, they could most likely be the result of evolutionary pressure driving the virus to change specific antibody binding sites. In this study we have shown that the number of mutations in Omicron and its subvariants is highly skewed towards an N to K substitution and that this characteristic is typical for Omicron and its subsequently emerged and dominant subvariants, solely occurs in the Spike protein region. Furthermore, our studies also show that altogether these mutations may potentially contribute to differences in stochastic movements (up *vs* down) and that the N to K mutation bias may potentially contribute to the alteration of RBD conformation. This type of mutation should therefore be considered in future vaccine design.

## Data availability statement

The original contributions presented in the study are included in the article/**Supplementary Material**. Further inquiries can be directed to the corresponding author.

## Author contributions

JB wrote first draft of the paper. JB and MP contributed with conceptualization of paper, data generation, writing and editing. QP, T-BN, DA and JH contributed with data generation, text editing. All authors contributed to the article and approved the submitted version.
