# Supplementary figures and images for: A bias of Asparagine to Lysine mutations in SARS-CoV-2 outside the receptor binding domain affects protein flexibility
Source: Front Immunol. 2022 Dec 9;13:954435. doi: 10.3389/fimmu.2022.954435 (PMC9788125; doi:10.3389/fimmu.2022.954435)

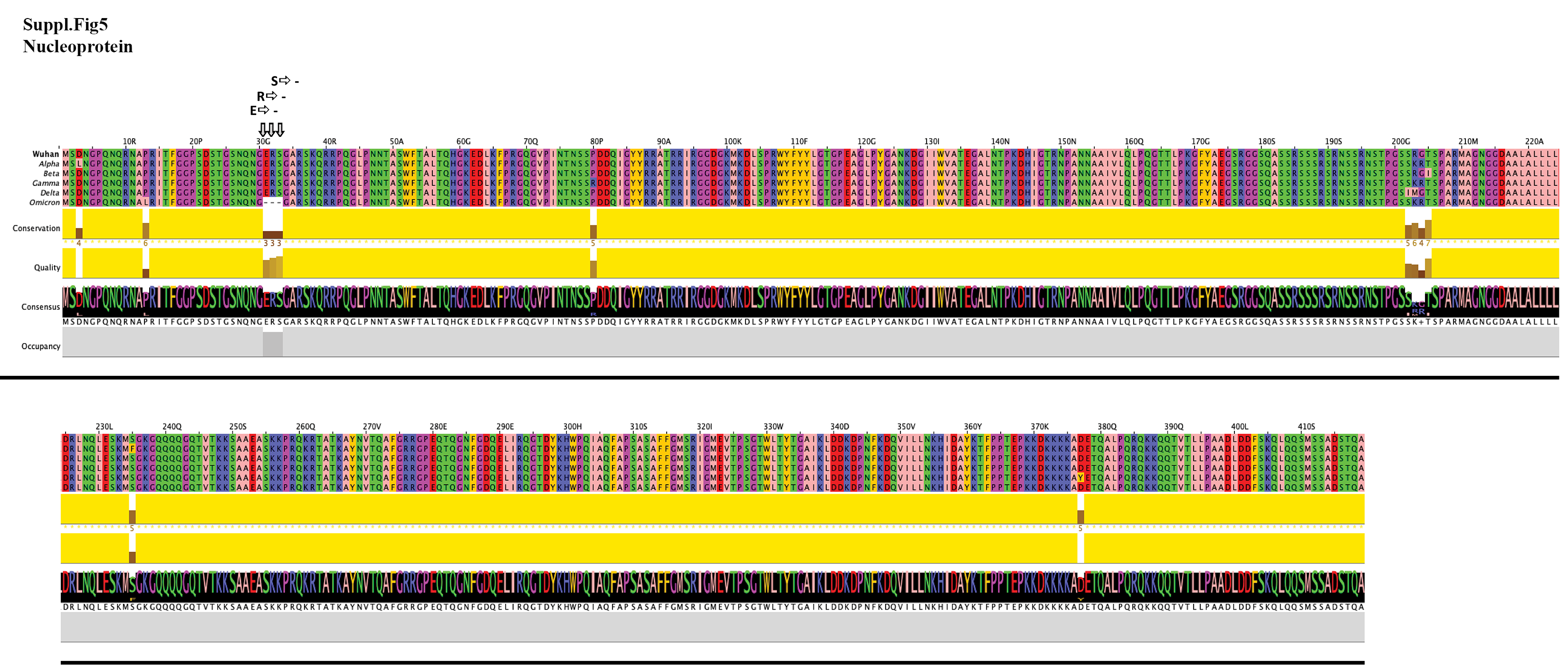

Supplement: Supplementary file 1 [file DataSheet_1.zip › JCB_etAl_Suppl/JCB_et_all_SupplFig5_nucleoprotein.tif]

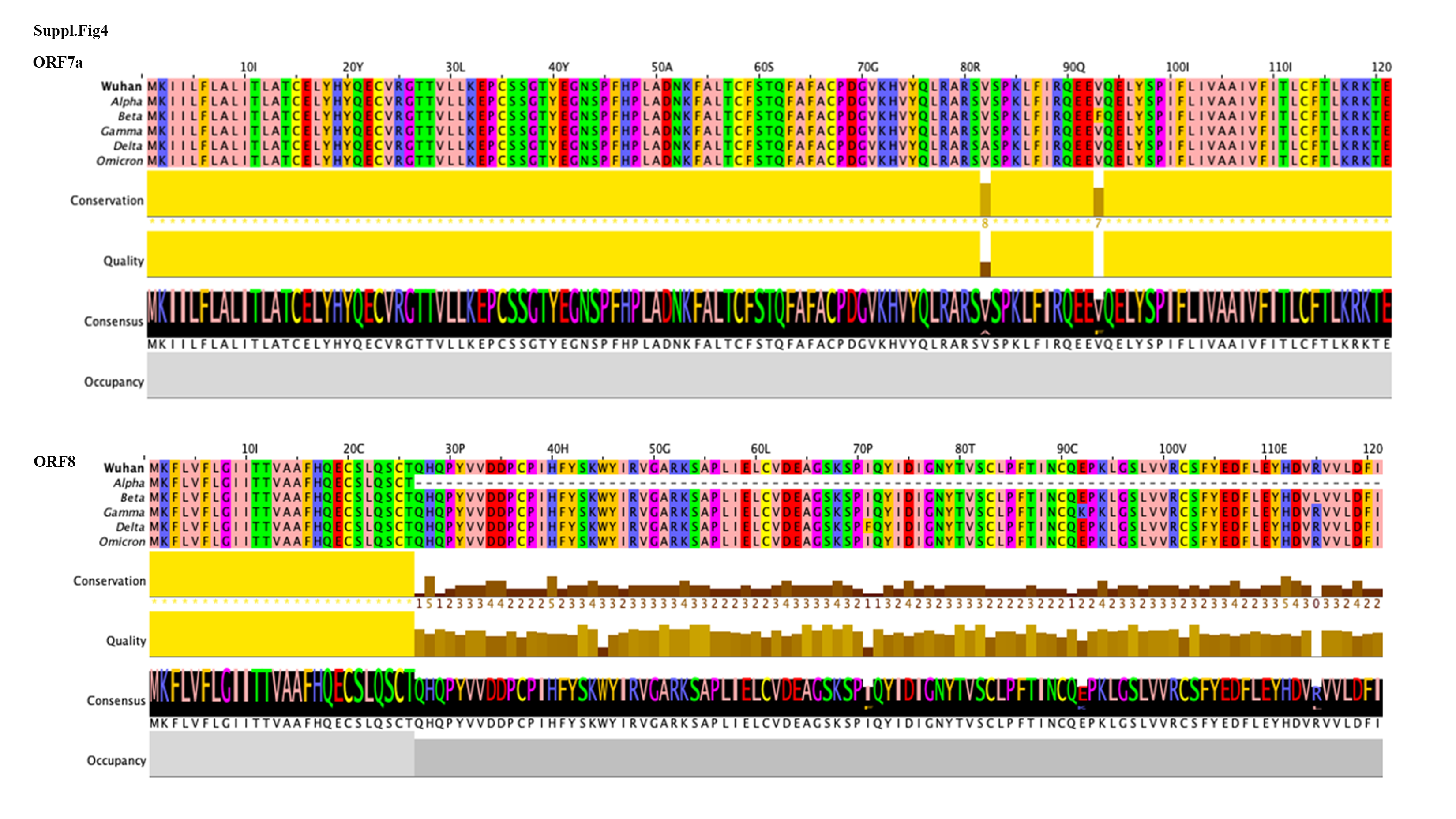

Supplement: Supplementary file 1 [file DataSheet_1.zip › JCB_etAl_Suppl/JCB_et_all_SupplFig4_ORF7aORF8.tif]

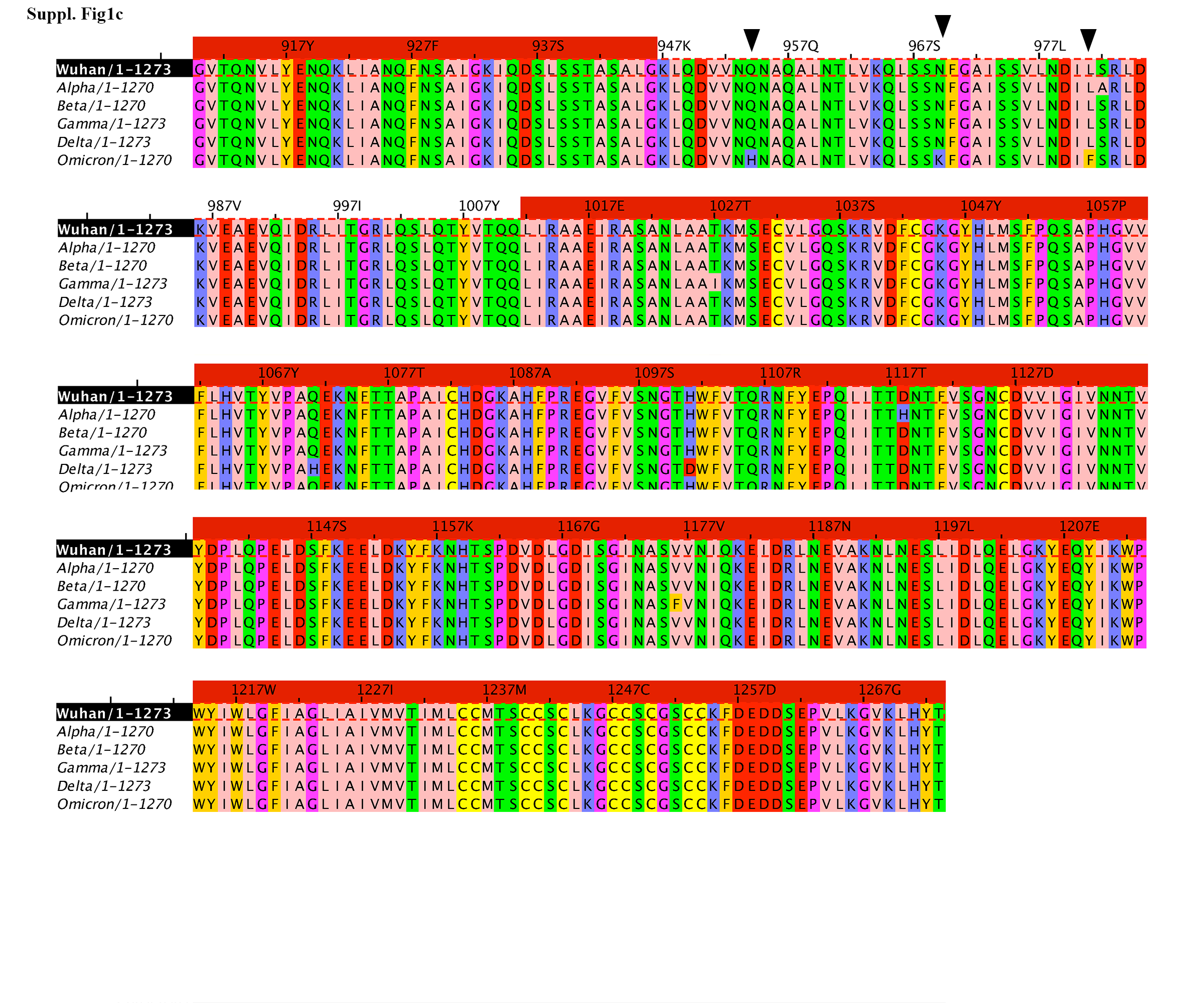

Supplement: Supplementary file 1 [file DataSheet_1.zip › JCB_etAl_Suppl/JCB_et_all_SupplFig1c_wholespike.tif]

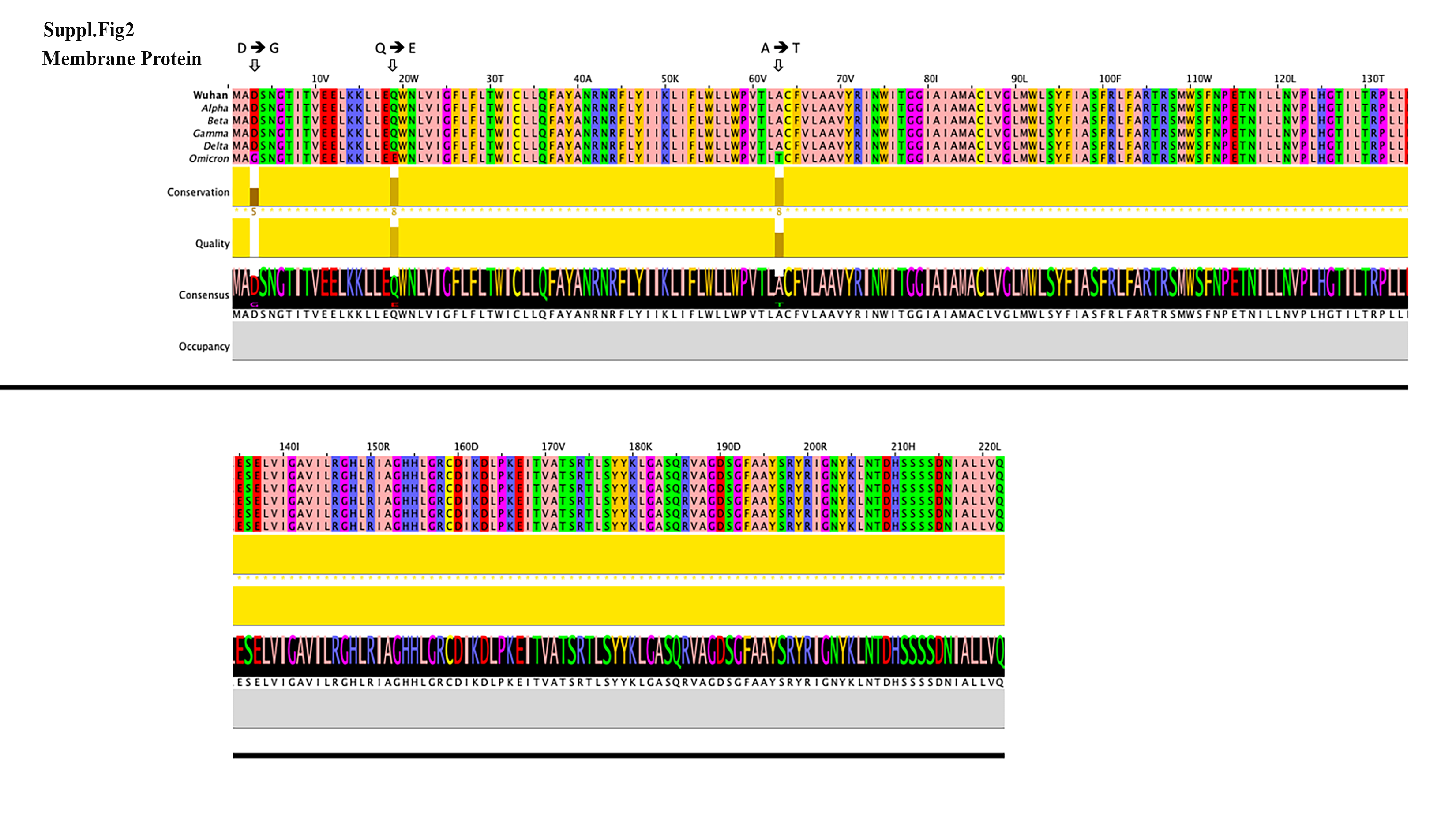

Supplement: Supplementary file 1 [file DataSheet_1.zip › JCB_etAl_Suppl/JCB_et_all_SupplFig2_membrane_protein.tif]

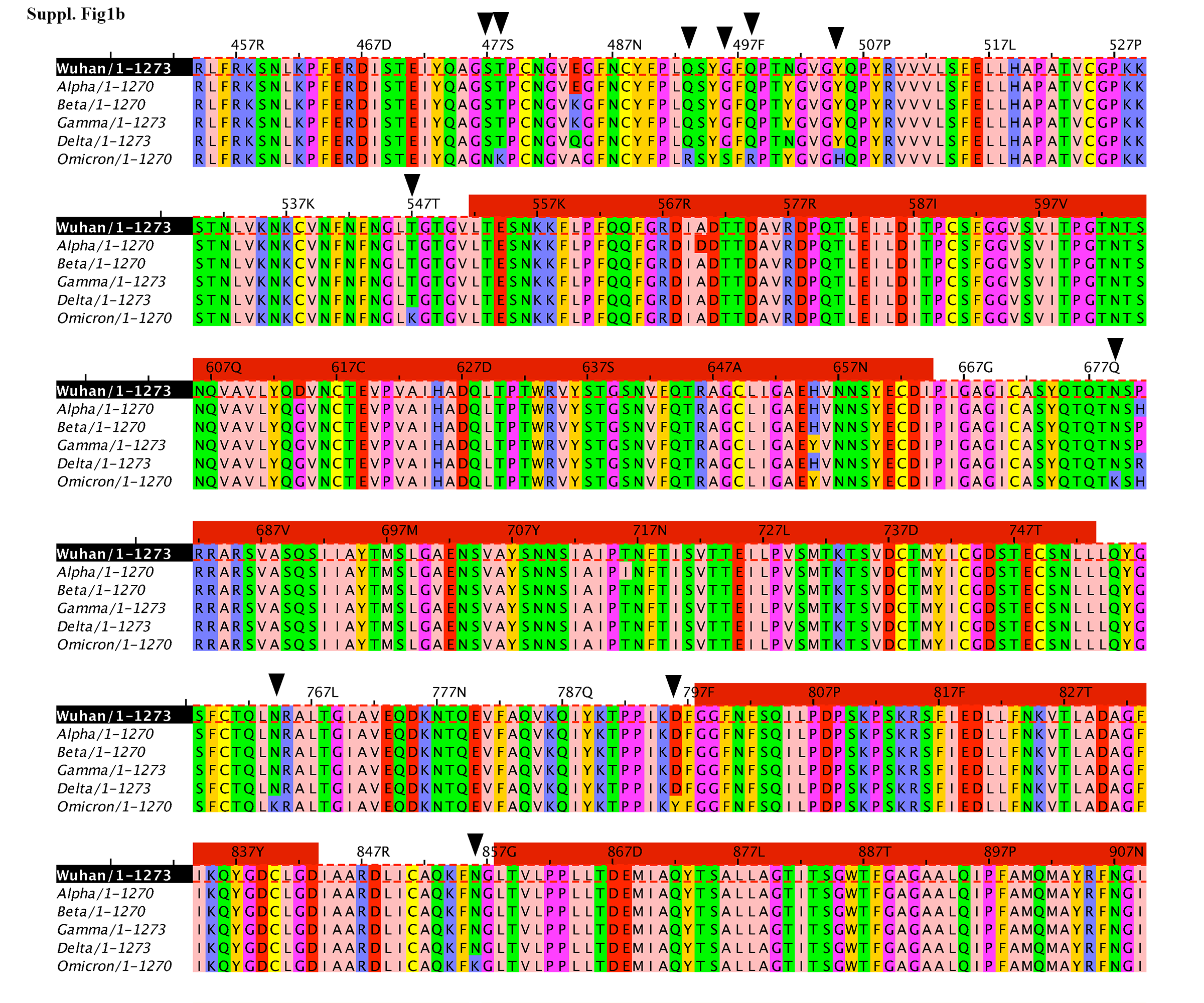

Supplement: Supplementary file 1 [file DataSheet_1.zip › JCB_etAl_Suppl/JCB_et_all_SupplFig1b_wholespike.tif]

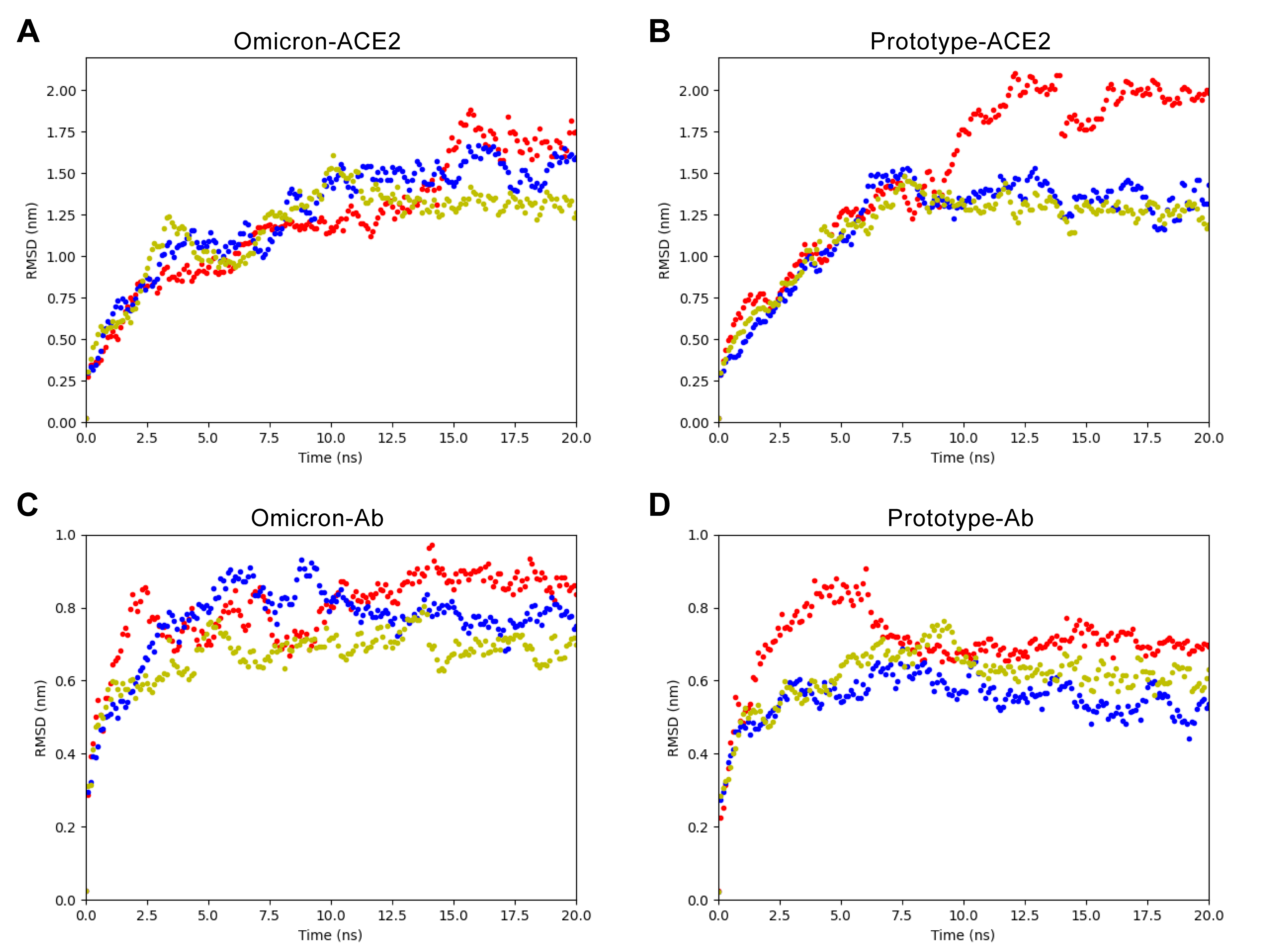

Supplement: Supplementary file 1 [file DataSheet_1.zip › JCB_etAl_Suppl/JCB_et_all_SupplFig6.tif]

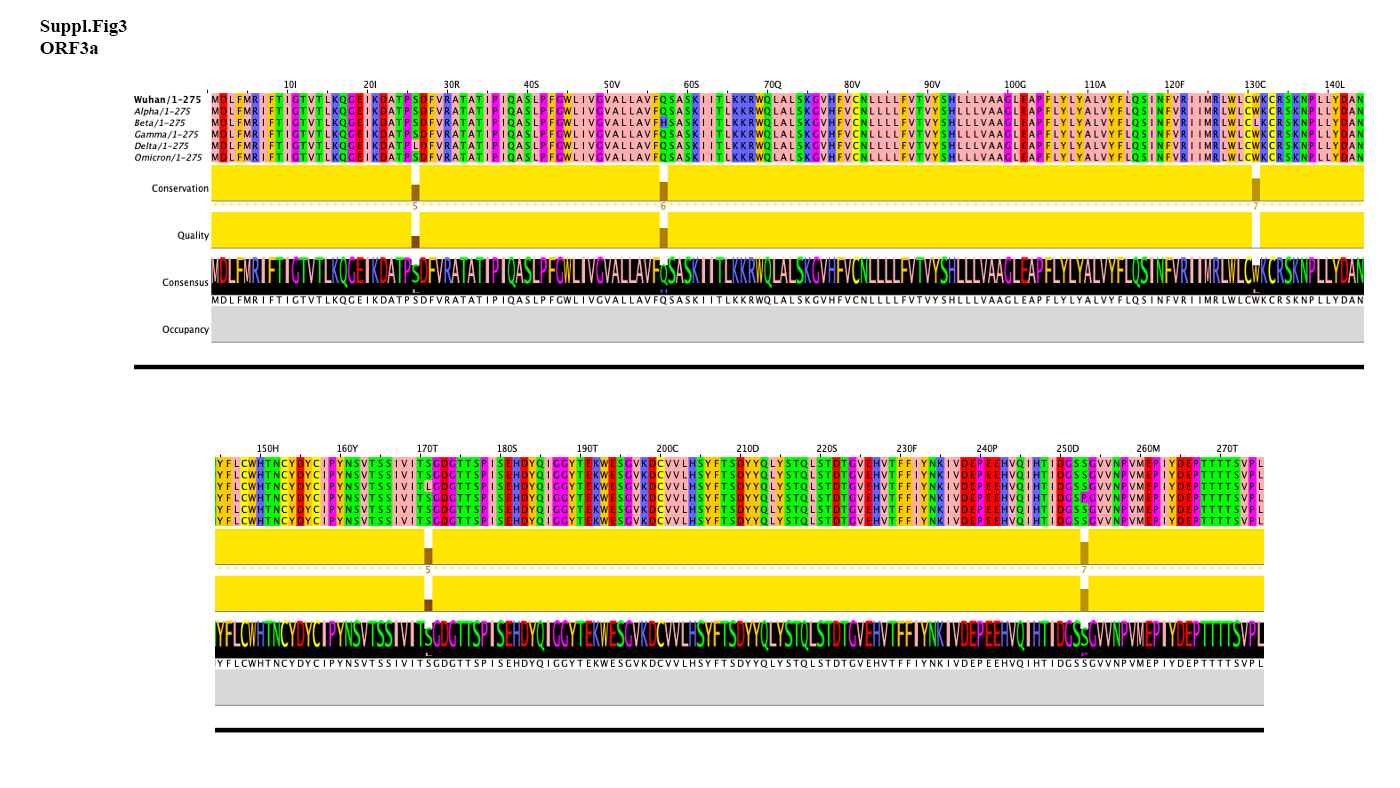

Supplement: Supplementary file 1 [file DataSheet_1.zip › JCB_etAl_Suppl/JCB_et_all_SupplFig3_ORF3a.tif]

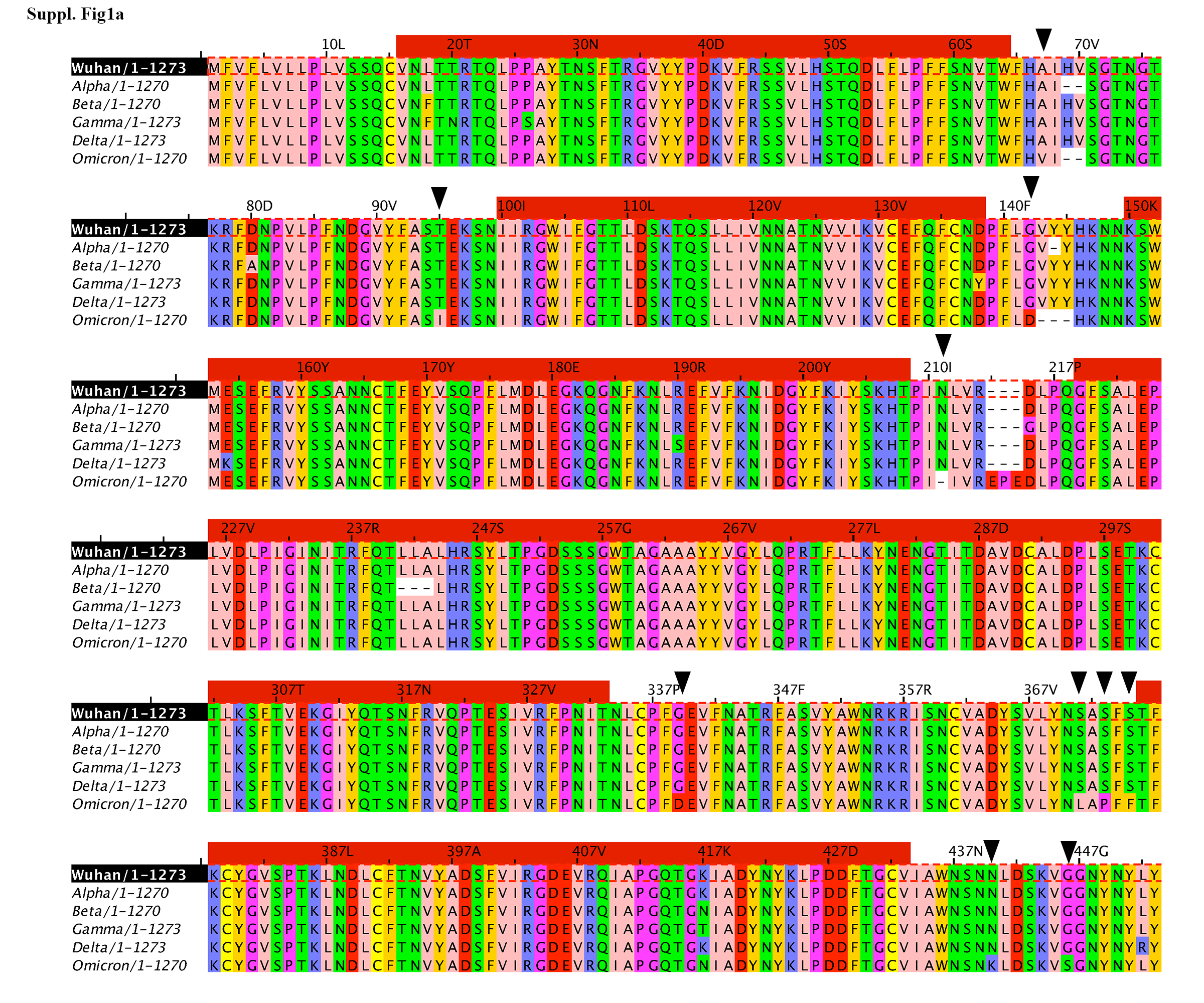

Supplement: Supplementary file 1 [file DataSheet_1.zip › JCB_etAl_Suppl/JCB_et_all_SupplFig1a_wholespike.tif]
