# Supplementary material for: A bias of Asparagine to Lysine mutations in SARS-CoV-2 outside the receptor binding domain affects protein flexibility
Source: Front Immunol. 2022 Dec 9;13:954435. doi: 10.3389/fimmu.2022.954435 (PMC9788125; doi:10.3389/fimmu.2022.954435)
Supplement: Supplementary file 2 [file DataSheet_2.docx]

**Mutations in SARS-CoV-2 Spike outside the RBD domain affect protein flexibility with implications for Omicron infectiousness and immune evasion**

Jennifer C. Boer^1^, Qisheng Pan^56^, Jessica K Holien^234^, Thanh-Binh Nguyen^56^, David B. Ascher^56^, Magdalena Plebanski^1^

^1^ School of Health and Biomedical Science, Royal Melbourne Institute of Technology, Melbourne, VIC 3000, Australia

^2^ Department of Medicine (St. Vincent's), University of Melbourne, Fitzroy, VIC 3065, Australia.

^3^ School of Science, RMIT University, Melbourne, VIC 3001, Australia.

^4^ Structural Biology Unit, St. Vincent's Institute of Medical Research, Fitzroy, VIC 3065, Australia.

^5^ School of Chemistry and Molecular Biosciences, University of Queensland, Brisbane City, QLD 4072, Australia.

^6^ Computational Biology and Clinical Informatics, Baker Heart and Diabetes Institute, Melbourne, VIC 3004, Australia.

**Table S1.** Missense tolerance ratio (MTR) retrieved from COVID-3D of all 30 missense mutations reported in Omicron variants.

| **Mutation** | **Relative solvent accessibility (RSA)** | **MTR** | **Solvent^*^** | **Outcome^*^** |
| --- | --- | --- | --- | --- |
| A67V | 0.00 | 0.69 | buried | intolerant |
| T95I | 0.02 | 0.78 | buried | tolerant |
| G142D | 0.39 | 0.90 | exposed | tolerant |
| N211I | 1.00 | 0.96 | exposed | tolerant |
| G339D | 0.61 | 0.61 | exposed | tolerant |
| S371L | 0.36 | 0.61 | exposed | tolerant |
| S373P | 0.67 | 0.62 | exposed | tolerant |
| S375F | 0.54 | 0.60 | exposed | tolerant |
| K417N | 0.30 | 0.68 | exposed | tolerant |
| N440K | 0.92 | 0.59 | exposed | tolerant |
| G446S | 0.77 | 0.55 | exposed | intolerant |
| S477N | 1.00 | 0.85 | exposed | tolerant |
| T478K | 0.39 | 0.83 | exposed | tolerant |
| E484A | 0.34 | 0.90 | exposed | tolerant |
| Q493R | 0.08 | 0.92 | buried | tolerant |
| G496S | 0.12 | 0.96 | buried | tolerant |
| Q498R | 0.20 | 0.99 | buried | tolerant |
| N501Y | 0.04 | 0.91 | buried | tolerant |
| Y505H | 0.15 | 0.82 | buried | tolerant |
| T547K | 0.41 | 0.88 | exposed | tolerant |
| D614G | 0.64 | 0.75 | exposed | tolerant |
| H655Y | 0.61 | 0.73 | exposed | tolerant |
| N679K | 0.94 | 0.67 | exposed | tolerant |
| P681H | 0.51 | 0.68 | exposed | tolerant |
| N764K | 0.25 | 0.99 | exposed | tolerant |
| D796Y | 0.48 | 0.98 | exposed | tolerant |
| N856K | 0.15 | 1.02 | buried | tolerant |
| Q954H | 0.27 | 0.81 | exposed | tolerant |
| N969K | 0.35 | 0.67 | exposed | tolerant |
| L981F | 0.24 | 0.54 | exposed | intolerant |

* Solvent exposure and missense tolerance were determined by using the cutoff determined in (1). The cutoff for missense tolerance was transferred from human study.

**Table S2.** Single-site mutation effects of the thermodynamics stability on prototype Spike protein.

| **RBD form** | **Protomer ID** | **Mutation** | **mCSM-Stability (Kcal/mol)** | **SDM (Kcal/mol)** | **DUET (Kcal/mol)** | **ENCoM (Kcal/mol)** | **DynaMut1 (Kcal/mol)** | **DynaMut2 (Kcal/mol)** | **mCSM-membrane (Kcal/mol)** |
| --- | --- | --- | --- | --- | --- | --- | --- | --- | --- |
| open | A | N764K | -0.21 | 0.61 | 0.39 | -0.33 | -0.36 | -0.62 | 0.67 |
| open | B | N764K | -0.14 | 0.81 | 0.50 | -0.27 | -0.32 | -0.50 | -0.25 |
| open | C | N764K | -0.14 | 0.38 | 0.41 | 0.22 | 0.94 | -0.47 | -0.17 |
| open | A | N856K | 0.02 | 0.38 | 0.57 | 0.49 | 1.32 | 0.80 | 0.49 |
| open | B | N856K | 0.02 | 0.03 | 0.33 | 0.73 | 1.94 | 0.93 | 0.42 |
| open | C | N856K | -0.04 | 0.03 | 0.27 | 0.23 | 0.64 | 0.02 | 0.63 |
| open | A | N969K | 0.06 | -0.76 | 0.24 | -0.32 | 0.00 | -0.95 | 0.50 |
| open | B | N969K | 0.05 | -0.93 | 0.20 | 0.17 | 0.61 | -0.96 | 0.74 |
| open | C | N969K | -0.03 | -0.55 | 0.21 | -0.06 | 0.22 | -0.98 | 0.34 |
| close | A | N764K | -0.26 | 0.53 | 0.32 | -0.04 | 0.03 | -0.60 | 0.26 |
| close | B | N764K | -0.23 | 0.20 | 0.27 | -0.13 | -0.37 | -0.63 | 0.26 |
| close | C | N764K | -0.28 | 0.66 | 0.33 | -0.06 | -0.21 | -0.92 | 0.25 |
| close | A | N856K | 0.07 | -1.32 | 0.05 | 0.23 | 0.27 | -0.39 | 0.61 |
| close | B | N856K | 0.06 | -1.00 | 0.12 | 0.67 | 1.06 | -0.46 | 0.34 |
| close | C | N856K | 0.00 | 0.03 | 0.31 | 0.48 | 1.30 | -0.66 | 0.50 |
| close | A | N969K | 0.09 | -0.62 | 0.18 | -0.54 | -0.08 | -0.93 | 0.61 |
| close | B | N969K | 0.05 | -0.50 | 0.17 | -0.26 | -0.06 | -0.99 | -0.51 |
| close | C | N969K | 0.05 | -0.62 | 0.13 | -0.29 | -0.57 | -0.99 | -0.46 |

**Table S3.** Single-site mutation effects of the interaction change among protomers on prototype Spike protein.

| **RBD form** | **Protomer ID** | **Mutations** | **mCSM-PPI (Kcal/mol)** | **mCSM-PPI2 (Kcal/mol)** | **Outcome** |
| --- | --- | --- | --- | --- | --- |
| open | A | N764K | -0.20 | -0.33 | Decreased affinity |
| open | B | N764K | 0.10 | -0.45 | Neutral |
| open | C | N764K | 0.15 | -0.43 | Neutral |
| open | A | N856K | -0.06 | -0.80 | Decreased affinity |
| open | B | N856K | -0.54 | -0.90 | Decreased affinity |
| open | C | N856K | 0.15 | -0.64 | Neutral |
| open | A | N969K | -2.45 | -0.21 | Decreased affinity |
| open | B | N969K | -2.41 | -0.30 | Decreased affinity |
| open | C | N969K | -2.54 | -0.25 | Decreased affinity |
| close | A | N764K | 0.12 | -0.20 | Neutral |
| close | B | N764K | -0.08 | -0.36 | Decreased affinity |
| close | C | N764K | -0.06 | -0.43 | Decreased affinity |
| close | A | N856K | -0.80 | -0.46 | Decreased affinity |
| close | B | N856K | -1.00 | -0.69 | Decreased affinity |
| close | C | N856K | -0.30 | -0.66 | Decreased affinity |
| close | A | N969K | -1.16 | -0.25 | Decreased affinity |
| close | B | N969K | -1.06 | -0.16 | Decreased affinity |
| close | C | N969K | -1.58 | -0.37 | Decreased affinity |


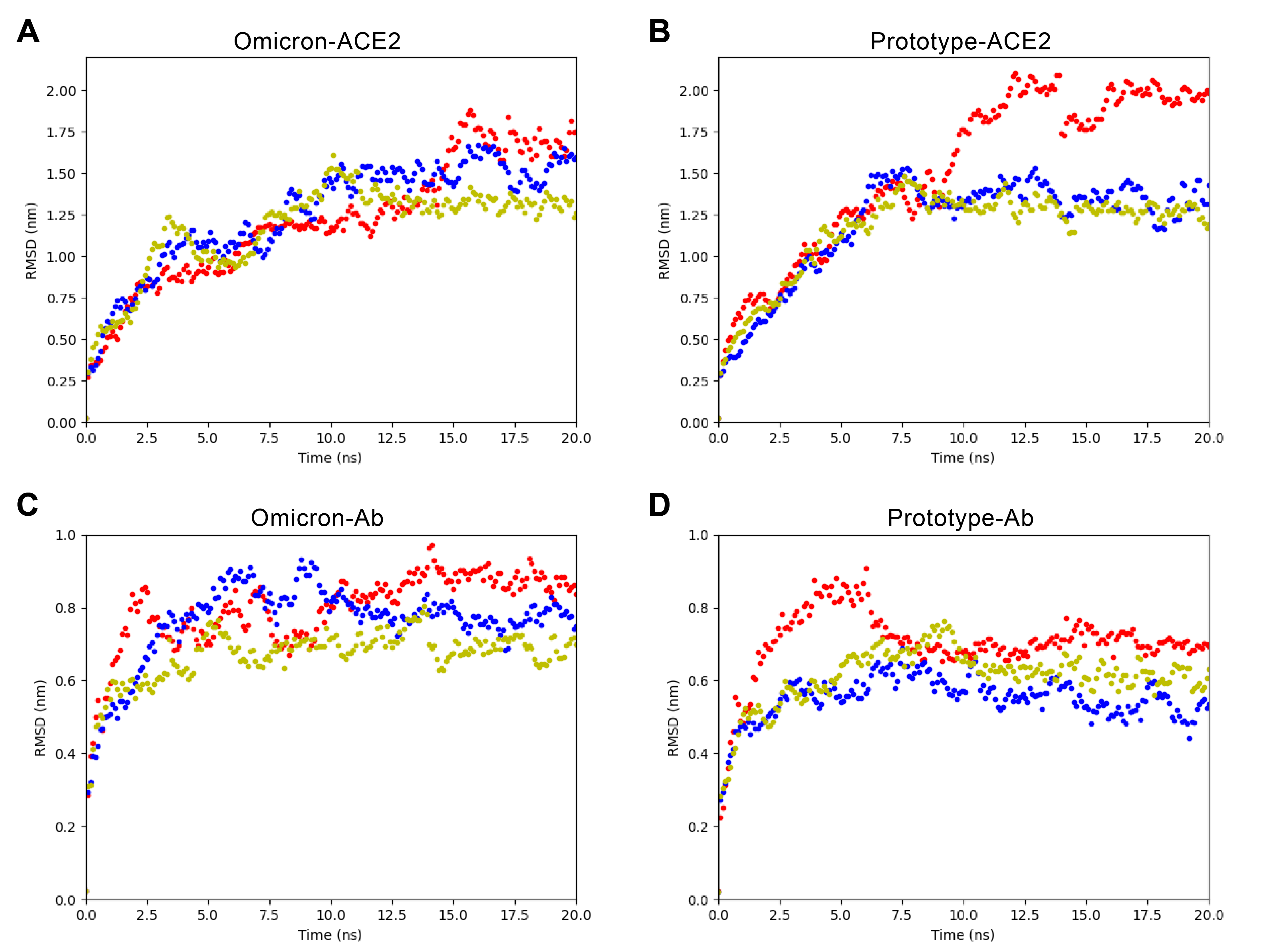


**Figure S6.** C-alpha Root mean squared deviation (RMSD) of Molecular Dynamics (MD) simulations of (A) Omicron-ACE2, (B) Prototype-ACE2, (C) Omicron-Ab, and (D) Prototype-Ab complex. Three 20 ns replicas of simulations were implemented, representing with blue, red, and yellow colours, respectively.

1. Silk M, Pires DEV, Rodrigues CHM, D’Souza EN, Olshansky M, Thorne N, et al. MTR3D: identifying regions within protein tertiary structures under purifying selection. Nucleic Acids Res. 2021 Jul 2;49(W1):W438–45.
